# Research progress on the application of 16S rRNA gene sequencing and machine learning in forensic microbiome individual identification

**DOI:** 10.3389/fmicb.2024.1360457

**Published:** 2024-02-02

**Authors:** Mai-Qing Yang, Zheng-Jiang Wang, Chun-Bo Zhai, Li-Qian Chen

**Affiliations:** ^1^Department of Pathology, Weifang People's Hospital (First Affiliated Hospital of Shandong Second Medical University), Weifang, China; ^2^Department of Second Ward of Thoracic Surgery, Weifang People's Hospital (First Affiliated Hospital of Shandong Second Medical University), Weifang, China

**Keywords:** forensic microbiome, individual identification, 16S rRNA, machine learning, review

## Abstract

Forensic microbiome research is a field with a wide range of applications and a number of protocols have been developed for its use in this area of research. As individuals host radically different microbiota, the human microbiome is expected to become a new biomarker for forensic identification. To achieve an effective use of this procedure an understanding of factors which can alter the human microbiome and determinations of stable and changing elements will be critical in selecting appropriate targets for investigation. The 16S rRNA gene, which is notable for its conservation and specificity, represents a potentially ideal marker for forensic microbiome identification. Gene sequencing involving 16S rRNA is currently the method of choice for use in investigating microbiomes. While the sequencing involved with microbiome determinations can generate large multi-dimensional datasets that can be difficult to analyze and interpret, machine learning methods can be useful in surmounting this analytical challenge. In this review, we describe the research methods and related sequencing technologies currently available for application of 16S rRNA gene sequencing and machine learning in the field of forensic identification. In addition, we assess the potential value of 16S rRNA and machine learning in forensic microbiome science.

## 1 Introduction and background

Forensic medicine represents a field that applies technologies from multiple disciplines such as medicine, biology, chemistry and physics to provide information for use in criminal investigations and evidence for trials, as well as a basis for human ethics and legislation (Atreya et al., 2022; Nteziryayo and Xinshe, 2023). A variety of assays including genetics, immunology, molecular biology and analytical chemistry have been utilized in forensic medicine to assess variations among microorganisms and speculate on specific microbial sources, relationships and transmission pathways (Haarkötter et al., 2021; Kumari et al., 2022).

Individual identification represents a fundamental component when gathering forensic evidence. Currently, this procedure mainly relies on DNA fingerprinting technologies and short tandem repeat (STR) composite amplification detection technology (Gouello et al., 2021). Microorganisms, which are present in the human body, are stable for a considerable period of time, can be found throughout the body and are present in any habitat involving humans. Accordingly, they can play a prominent role in individual identification (Haarkötter et al., 2021). One genetic component which may serve as a critical marker for this procedure is the16S rRNA, which is a small subunit of ribosomal RNA and the coding gene for 16S rDNA. As DNA is easy to extract and relatively stable, 16S rDNA has become the main marker for use in sequencing amplification to then identify phylogenetic relationships. After sequencing, 16S regions can be analyzed bioinformatically (Song et al., 2018). Currently, with the development of high-throughput sequencing (HTS), it has been possible to use 16S rRNA gene sequencing in forensic microbiome analyses for the identification of individuals (Cao et al., 2021). Moreover, when combined with machine learning, a primary artificial intelligence (AI) technique employed in forensic research, deep insights into microbial information analysis, as related to 16S rRNA gene sequencing, can be achieved (Ghannam and Techtmann, 2021).

## 2 Review

### 2.1 Current status of forensic microbiomes for use in individual identification

Microorganisms, a class of simple organisms of modest evolutionary status, are considered to be at the initial stages of complex evolution and thus represent the most primitive form of life in the biosphere. Microorganisms, which have recently been deemed as potentially useful in criminal investigations, are widely used in industrial, agricultural, and pharmaceutical production, and are intimately related to human life (Metcalf et al., 2017). Acquiring bioinformation through inspection and identification is an important element of any criminal investigation (Haarkötter et al., 2021; Kumari et al., 2022). Microbiology has played a role in these forensic investigations, mainly through its use in focusing on soil samples and human and dead microorganisms (Szelecz et al., 2018; Yuan et al., 2023).

Identifying the source of biological evidence is one of the fundamental goals of forensic genetics as it can be used to identify bodies and suspects, as well as track the biological stages associated with a crime (Cho and Eom, 2021; Gouello et al., 2021; Baliso et al., 2023). While the methods used for detecting human DNA continue to advance and show increased levels of sensitivity, not all DNA samples are suitable for current methods. For example, with outdated, severely degraded or decayed forensic materials, multiple nuclear gene based species identification methods are often considered to be insufficient with regard to their feasibility and accuracy in identification (Sherier et al., 2022). In contrast, the large number of microorganisms in the skin, mucous membranes and cavities that coexist within the human body and come into contact with the environment are relatively stable under normal circumstances. In the process of their long-term evolution, these microorganisms depend on, and constrain, each other in the human body, forming a dynamic microecological equilibrium that remains stable despite drastic changes that occur in the external environment. As a result of the differences present among individuals due to variations in their professions and lifestyles, different individuals may form unique microbial fingerprints. Such fingerprints may then provide a theoretical basis for tracking the origin of these microorganisms in forensic microbiology, and thus can be analyzed to achieve individual identifications (Guleria et al., 2023). The human microbiome, which encompasses the fungi, bacteria and viruses living in and on individuals and their surrounding environment, contributes significantly to the genetic content and, in this way, is unique to each individual. Compared to human cells, microorganisms offer the advantages of being present in large quantities and are more stable. Their shedding, transfer and deposition are similar to that of cells, but their greater numbers and stability make them better candidates for use in the detection of physical evidence. In fact, results from previous studies have demonstrated the potential to use microbiome profiling for forensic applications (Schmedes et al., 2017; Wang et al., 2022a,b).

In 2019, Woerner et al. reported that microbial strain composition is more individualized than that of a phylogeny, suggesting that microbial composition may be more effective in recognizing individuals than that of recent common ancestry. One inference from these findings is that host-environment interactions may maintain a targeted microbial profile which may not necessarily be repopulated by intra-individual microbial strains (Woerner et al., 2019). As an approach to test the potential for the bacterial and fungal microbiome diversity within the soil to be used as a legitimate source of evidence in the resolution of homicide cases, a total of 12 soil samples were collected, including two evidence samples, three crime scene samples and seven non-crime scene related control samples. The results of this study indicated that the distance between evidence samples and crime scene reference samples was closer to each other than that of the non-crime scene related control samples. As based upon these results, it seems that bacterial, and especially fungal DNA, in the soil have the potential to provide an effective source of evidence for use in the resolution of forensic cases. In this way, microbiome analyses of soil samples obtained in homicide cases offer the possibility to establish a relationship between the case and the crime scene (Karadayı, 2021). Examining the microbial makeup of soil for the determination of its origin and differentiation of soil samples are well-established procedures. When assessed over time, soil samples stored open at room temperature were found to be more similar to soil from evidence samples as compared with that from soil samples stored bagged and/or frozen. Even with as little as 1 mg, evidence soil samples were found to be associated with the correct habitat 99% of the time, a finding which accentuates the importance and successful application of using *ex situ* microbial changes as forensic evidence (Foran and Badgley, 2020).

The human skin microbiome has recently been investigated as a potential forensic tool, an approach which offers a novel use of skin microbiomes (Neckovic et al., 2020; Tozzo et al., 2020). With use of nucleotide diversities of stable clade-specific markers with supervised learning it was possible to classify skin microbiomes from a particular individual with an up to 100% classification accuracy, as assayed from samples obtained at three different body sites. Attribute selection was used to identify 187 genetic markers from 12 clades which then provided the greatest differentiation among individual skin microbiomes from 14 skin sites. In this way, skin microbiome profiling from a supervised learning approach enables a high degree of classification accuracy for samples collected from individuals over a relatively long time period, a result which has an important application potential for use in forensic human identification (Schmedes et al., 2017). When comparing skin and surface microbiomes, Wilkins et al. (2021) found that a person could be accurately matched to their household in 84% of tests and to their neighborhood in 50% of tests and, this matching accuracy did not decay for household surfaces over the 10-day study period, although it did for samples from public surfaces. Interestingly, the time of day at which a skin or surface sample were obtained affected the matching accuracy. These results suggest that in addition to considering the decay curves of microbiota traces over time, diurnal patterns in microbiome acquisitions that contribute to the human skin microbiome assemblage represent important factors for consideration in the development of this as a potential forensic method (Wilkins et al., 2021). The human skin hosts a variety of microbes that can be transferred to surfaces (“touch microbiome”) which enables these microorganisms to be considered as forensic markers, similar to that of “touch DNA.” As a means to evaluate the transferability and persistence of the “touch microbiome” on a surface, the deposition of a fingerprint and its exposure from 11 volunteers were assessed from samples maintained at room temperature over a 30 day period. The results revealed that 6 skin core microbiome taxa were identified, as well as unique donor characterizing taxa. These unique taxa may have relevance for personal identification studies and may be useful to provide forensic intelligence information when “touch DNA” fails (Procopio et al., 2021).

The oral microbiome harbors microbial community signatures that also differ among individuals, highlighting the highly individualized information that can be garnered from these samples. Saliva, a common body fluid with significant forensic value, has been used in criminal investigations involving murder and assault (D'Angiolella et al., 2020). In 2016, Leake et al. investigated the potential for bacteria found in the salivary microbiome to be used as a means to differentiate individuals. Their results indicated that it was possible to distinguish between two people using the bacterial microbiota present in their saliva, regardless of time of sampling (Leake et al., 2016). Liang et al. established a prediction model based on the random forest algorithm that could distinguish saliva between different regions at the genus level. However, this model has a certain probability for error and thus requires more in-depth research. Nonetheless, the microbial community information in saliva samples have a potential for application in body fluid identification and biogeographic inference (Liang et al., 2022). Sundström et al. investigated the shared bacterial communities among family members and adult children and found that greater similarities were observed as related to mothers versus fathers. The observed similarity in oral microbiome between parent–child pairs seems to weaken over time. Taken together these results suggest that this approach was suitable for a relatedness study of multigenerational salivary bacteria microbiomes (Sundström et al., 2020).

Human gut microbiota are individually unique, indicating that microbiota in fecal traces left at a crime scene could act as a potential biomarker for forensic individual identification (Wang et al., 2022a,b). Males were found to be characterized by taxa in the phylum Proteobacteria, while females by the Synergistetes phylum. The gut bacterial community assembly mechanism was mainly affected by some process (sex, body mass index). Subjects with different individual characteristics have specific gut microbiota, and thus can be discriminated by bioinformatics methods, suggesting that an assay of gut microbiota can serve as a means for forensic personal identification (Wang et al., 2022a,b).

Collectively, the findings from these investigations reveal that the bacterial and fungal microbiome diversity within the soil, skin, saliva and gut microbiota represent significant sources that can be applied for use in forensic personal identification.

### 2.2 Introduction of related technologies using 16S rRNA gene sequencing and machine learning

Bacteria comprise one of the main groups of microorganisms and, within bacteria, there are three main types of ribosomal RNAs (rRNA), 5S, 16S, and 23S (Tsukuda et al., 2017). Among these, the 16S rRNA is a small subunit of bacterial ribosomal RNA and the coding gene for this subunit is 16S ribosomal DNA (rDNA). The total length of 16S rRNA is approximately 1,540 nt and it is present in the ribosomes of all bacteria. Due to its highly conserved structure and function, HTS is often used in microbial ecology research to determine its gene fragments (Tozzo et al., 2020). After sequencing, 16S regions may be analyzed bioinformatically. The degree of similarity in the sequencing reflects the remoteness of microbial phylogenetic relationships, while the abundance of corresponding microorganisms in the community is indicated by the number of sequence occurrences. In this way, it is possible to obtain information on the species composition ratio and diversity within microbial communities (Watts et al., 2017; Johnson et al., 2019; Hassler et al., 2022).

The 16S rRNA gene sequencing technique was first employed for use in phylogenetic analysis in 1985 (Lane et al., 1985). This sequence contained ten “highly conserved regions” for primer design and nine “hypervariable regions” which could then be used to identify phylogenetic characteristics of microorganisms. Conserved regions reflect the phylogenetic relationships among bacterial species and universal amplification primers can be designed as based on their sequences, while highly variable regions reflect the differences among bacterial species. Bacterial 16S rRNA genes contain nine “hypervariable regions” (V1–V9) that demonstrate considerable sequence diversity among different bacteria. Specifically, V1 best differentiates among *Staphylococcus aureus* and coagulase negative Staphylococcus sp., V2 and V3 can distinguish all bacterial species at the genus level, V6 can distinguish among most bacterial species except Enterobacteriaceae and V4, V5, V7, and V8 were found to be minimally effective as targets for genus or species-specific probes (Chakravorty et al., 2007). Universal primers can be designed based on their sequences and thus can serve to identify and classify bacteria. As 16 s rRNA can distinguish among different species, it plays an important role in bacterial taxonomy and development. Therefore, 16S rRNA gene sequencing has become the most widely used marker gene for profiling bacterial communities (Chakravorty et al., 2007; Yang et al., 2016; Song et al., 2018).

The first generation of sequencing technology, as represented by Sanger’s dideoxynucleotide chain termination method and Maxam’s chemical degradation method, were performed in 1977, and enabled the first complete genome sequence identification of bacteriophage phi X174 (Sanger et al., 1977). These technologies were collectively referred to as the first generation sequencing technology (Slatko et al., 2011). More recently, a series of second-generation HTS technologies, such as Roche’s 454 sequencing platform, Illumina’s SolexaGenomaAnalyzer platform and Applied Biosystems (ABI) Solid sequencing platform, have been developed (Hu et al., 2021; Meslier et al., 2022). As compared with that of the first generation sequencing technologies, the higher throughput of HTS has proved to be more effective for use in microbial genomics research and has quickly become the main detection method for microbial genomics (Budowle et al., 2014). At present, the main research entity in forensic microbiology is 16S rDNA. Procedures involved with the use of 16S rDNA include the extraction of microbial DNA, polymerase Chain Reaction (PCR) templates for high variability regions of 16S rDNA, library construction, template preparation, machine sequencing and bioinformatics analysis of sequencing data (Sanschagrin and Yergeau, 2014; Bador et al., 2020). HTS and genomic sequencing technologies have revolutionized the field of microbiology as they enable a detailed study of microorganisms, leading to a rapid expansion of biological data.

Advances in nucleic acid sequencing technology have enabled expansion of our ability to profile microbial diversity as it can provide a means for assessing the generation of microbial community profiles for hundreds and, even thousands of samples. Normally, it would be difficult to extract meaningful information from these large datasets, however, this problem has been largely resolved with the recent application of machine learning for this microbial analysis (Zhou and Gallins, 2019; Ghannam and Techtmann, 2021). In machine learning, a computer is supplied with a dataset and associated outputs. The computer then “learns” and generates an algorithm describing the relationship between the supplied dataset and associated outputs. By developing algorithms that best represent a set of data. The algorithm can be explicitly coded using known features, machine learning uses subsets of data to generate an algorithm that may use novel or different combinations of features and weights than can be derived from first principles. This algorithm can be used for inferences involving future datasets (Choi et al., 2020).

### 2.3 The application of 16S rRNA gene sequencing and machine learning in forensic microbiome individual identification

Microbiome research represents a highly transdisciplinary field encompassing a wide range of protocols associated with its use. Recent advances in molecular sequencing and computational techniques have significantly contributed to this field. For example, massive parallel sequencing (MPS) technology, also referred to as HTS, substantially improved the amount of sequencing data to be processed and surmounts the limitations of non-cultured bacteria to be sequenced, rendering this information available for forensic microbiome analysis (Tozzo et al., 2020). The 16S rRNA gene sequencing method has been applied in forensic microbiology, including identification of biological and soil samples, as well as providing inferences regarding postmortem interval (PMI), mechanisms of drug addiction and individual identification (Cao et al., 2021; Garg et al., 2021; Yang et al., 2022; Liu et al., 2023).

In forensic microbiome research, the 16S rRNA gene sequencing method has been used for the study of microorganisms in samples from saliva, skin and the gut (Soriano-Lerma et al., 2020; Rozas et al., 2021; Ibal et al., 2022). In 2012, the inter- and intra-individual variations in microbial communities from 264 saliva samples of 107 individuals were characterized using culture-independent 16S rRNA pyrosequencing. With this technique, individuals were found to be more similar to themselves and their co-twins in the 12–17 and 17–22 aged cohorts as compared to that of the entire population sample. An additional finding was that no statistically significant differences in similarity were obtained between monozygotic versus dizygotic twin pairs (Stahringer et al., 2012). When two different targets (16S rRNA and rpoB) were combined to maximize the analysis of the salivary microbiome, there was an increase in the power of differentiation. Streptococcus, a Firmicutes which is one of the most abundant aerobic genera found in saliva and targets Streptococcus rpoB, enhances the characterization among different streptococci species, an effect which cannot be differentiated using 16S rRNA alone. It was also observed that the individual identification of samples from the same group of people were maintained regardless of the time of sampling (Leake et al., 2016). This 16S rDNA sequencing technology was also used to sequence the V3-V4 hypervariable regions in saliva samples from five different cities in China (Guangdong, Qinghai, Henan, Zhejiang, and Jilin) to reveal the role of regional location on the heterogeneity of microbial profile information in saliva. These investigators were then able to establish a prediction model based on the random forest algorithm that could distinguish saliva samples as obtained between different cities at the genus level (Liang et al., 2022).

Widespread use of 16S rRNA gene sequencing has been applied for use in forensic microbiome individual identification. However, 16S rRNA or shotgun metagenomic sequencing, when used to characterize skin microbiomes, have limited species and strain resolution and a susceptibility for stochastic effects. The hidSkinPlex technique was initially tested for its capacity to evaluate three bacterial control samples. In 2018, all skin samples (*n* = 72), regardless of body site origin, were correctly classified with an accuracy of up to 94%, while body site origin could be predicted with an 86% accuracy. HidSkinPlex provides a novel, targeted enrichment approach to profile skin microbiomes for human forensic identification purposes (Schmedes et al., 2018). Clustered regularly interspaced short palindromic repeats (CRISPRs) represent prokaryotic genetic elements that can provide a history of infections encountered by the bacteria. The individual specificities, as identified using CRISPR typing, were confirmed by comparing the CRISPR diversity to microbiome diversity, as assessed using 16S rRNA amplicon sequencing. CRISPR typing achieved an accuracy of 95.2% in personal classification, whereas 16S rRNA gene sequencing only achieved an accuracy of 52.6%. These results suggest that sequencing CRISPRs in the skin microbiome may be a more powerful approach for use in personal identification and ecological studies as compared with that achieved using conventional 16S rRNA gene sequencing (Johnson et al., 2019).

When profile microbial diversity, we faced numbers of samples. It would be difficult to extract meaningful information from these large datasets, however, this problem has been largely resolved with the application of machine learning for this analysis. Various machine learning methods, including Random Forest, Support Vector Machines, Linear Regression and Logistic Regression also play a crucial role in forensic science (Handelman et al., 2018). Random Forest is a widely used machine learning method with good performance on classification and regression tasks. It works well under low sample size situations, which benefits applications in the field of biology (Tian et al., 2023). Support Vector Machine can be considered a special neural network, which is supervised learning method that can have different kernel functions for its decision function. The objective of the kernel method is to convert the original problem into a linearly solvable one. With its use, the data describing the problem to be solved are transformed into the kernel space through the application of nonlinear transformations (Dani et al., 2023). Linear and logistic regressions are widely used statistical methods to assess the association between variables in medical research. These methods estimate if there is an association between the independent variable and the dependent variable (Castro and Ferreira, 2023). And can be used in forensic medicine (Siino and Sears, 2020; Xi et al., 2022). In 2016, a sequencing of 16S rRNA and 18S rRNA genes was performed on soil samples near a corpse to extract microbial diversity characterization related to the decomposition of a body. Random forest regression and dynamic Bayesian networks were then used to assess the predictability of microbial succession in different soil types and host species (Metcalf et al., 2016). Tackmann et al. (2018) analyzed sequencing data from five human body regions and soil samples, then trained a random forest model with human body part classification performance, to identify the core set of biomarkers. This study represented the first time AI was used to identify specific microbial biomarkers within a human body part. In 2021, single-nucleotide polymorphisms with the highest Wright’s fixation index estimates were then selected for predicting donor identity using a support vector machine learning model. Three different single-nucleotide polymorphisms selection criteria were employed: single-nucleotide polymorphisms with the highest Wright’s fixation index estimates (i) common between any two samples regardless of markers present (termed *overall*); (ii) each marker common between samples (termed *per marker*); and (iii) common to all samples used to train the machine learning algorithm for human identification (termed *selected*). The single-nucleotide polymorphisms chosen based on criteria for *overall*, *per marker*, and *selected methods* resulted in an accuracy of 92.00, 94.77, and 88.00%, respectively. The results support that estimates of Wright’s fixation index, combined with machine learning, can notably improve forensic human identification via skin microbiome profiling (Sherier et al., 2021). Accordingly, it signified a new scientific and technological method for use in forensic microbial individual identification surveys.

## 3 Challenges and the way forward

In forensic science, information derived from microorganisms are highly valued due to their diversity and ubiquitous nature. Moreover, specific microbial communities are often closely related to their environment, and to the changes in processes to which they are subjected (Procopio et al., 2021). From reports available in the literature, 16S rRNA gene sequencing, as obtained from saliva, skin and gut samples, have been frequently used in forensic microbiome individual identification (Soriano-Lerma et al., 2020; Rozas et al., 2021; Ibal et al., 2022).

The 16S rRNA gene sequencing method has also been applied in forensic microbiology, including identification of biological and soil samples along with inferences regarding PMI, mechanisms of drug addiction and, as noted above, individual identification (Cao et al., 2021; Garg et al., 2021; Yang et al., 2022; Liu et al., 2023). Not surprisingly, the unique advantages of 16S rRNA sequencing in microbial genomics for use in individual identification have become a cutting-edge topic of considerable interest in forensic research. However, it is also important to acknowledge some of the deficiencies and limitations in the practical application of 16S rRNA gene sequencing. For example, while metagenomics focuses on the overall microbial community, including bacteria, fungi and archaea, 16S rRNA gene sequencing only analyzes the structure of bacterial communities.

Although 16S rRNA gene sequencing has great potential for assessing individual identification in forensic microbiology, there remain insufficient data to establish a universal applicability and accuracy of this method for individual identifications and inferences. The wide variety of microorganism types, the complexities of interactions among microbial communities and variations in their distribution among different individuals and environments represent assets, but also current potential liabilities, associated with this technique. To improve upon the analysis of data as accumulated using 16S rRNA gene sequencing, forensic microbiology experiments, sample collection, DNA extraction methods and standards for recording environmental data need to be unified to eliminate errors that can result from different operators or operating methods. In this way, each sample can objectively and reliably reflect the microbial community structure to provide a comprehensive comparative analysis of experimental data from cadavers under the varied condition in which these samples were collected. At this time, individual identification in forensic microbiology is in its early stages of development and assessment, and much work remains before it can become a universally accepted procedure. Some notable issues to be addressed will be to establish a system of forensic microbiological assays for individual identification, evidence purification, evidence collection and preservation, evidence identification and analysis, and the application of information as obtained from AI technology data.

The AI technology resulting from machine learning has been widely applied in face recognition, DNA electrophoresis analysis, and individual recognition due to its unique advantages in image recognition. In the field of forensic science, although there has been rapid development in the application research of AI, there is still controversy over whether it can be extended to practical applications in forensic medicine. In addition to ethical challenges brought about by new technologies, there are also many issues, such as how to choose appropriate algorithms, how to choose analysis platforms, and how to determine application standards, and so on. There is little doubt that machine learning will also serve as a significant tool for further advances in the field of forensic research.

## 4 Conclusion

In summary, 16S rRNA gene sequencing is widely used in forensic microbial identification of individuals. Employing microbial communities as research targets and assaying their base level of conservation can provide valid and reliable information reflecting the species condition with a high degree of accuracy. Compared to traditional methods, 16S rRNA gene sequencing has the capacity to provide rapid, accurate and valuable information regarding individual identifications. Moreover, the cost of this technique is lower than that of metagenomic sequencing. With the inevitable development of novel technologies, many of the complexities and challenges associated with forensic microbiology will be resolved. In specific, AI can help forensic appraisers extract comprehensive patterns and useful information from vast and complex data, maximizing the value of these data, and thus enhance the application of forensic microbiology to enable microbial identification become a new driving force in the development of forensic science. In this way, the technology as based on AI and automated information technology, provides a convenient, accurate and repeatable method for forensic science. In the near future, we hope to expand upon the collection of human body samples and conduct more in-depth research using machine learning algorithms for application in forensic practice.

## Author contributions

M-QY: Writing – original draft, Writing – review & editing. Z-JW: Data curation, Methodology, Writing – original draft. C-BZ: Data curation, Methodology, Writing – original draft. L-QC: Writing – original draft, Writing – review & editing.
